# Why hasn't the National Institute been ‘NICE’ to patients with colorectal cancer?

**DOI:** 10.1038/sj.bjc.6600369

**Published:** 2002-06-05

**Authors:** M P Saunders, J W Valle

**Affiliations:** Christie Hospital, Wilmslow Road, Manchester M20 4BX, UK

## Abstract

*British Journal of Cancer* (2002) **86**, 1667–1669. doi:10.1038/sj.bjc.6600369 www.bjcancer.com

© 2002 Cancer Research UK

## 

On the 1st of April 1999, the National Institute of Clinical Excellence (NICE) was established to evaluate the clinical and cost effectiveness of various different medical technologies including pharmaceuticals. Initially, a technology is assessed, after which provisional and then final advisory documents (PAD and FAD) are produced prior to the publication of clinical guidance with particular attention being paid to high quality adequately powered Randomised Controlled Trials (RCT's). Interested parties can submit evidence at the start of the process and can then respond to the provisional guidance released as part of the PAD. They can also appeal after the final guidance is produced, but if this appeal is unsuccessful, the process stops and the ‘Technology Appraisal Guidance (TAG)’ is published on the NICE web site and as a booklet.

The guidance on the use of irinotecan, oxaliplatin and raltitrexed for the treatment of advanced colorectal cancer (CRC) was published in March 2002 (NICE, 2002). The process began in August 2000 and reports from the pharmaceutical industry, UKCCCR, CancerBACUP and Macmillan Cancer relief were submitted by December of that year. Advice was also sought from the MRC trials unit, Royal College of General Practitioners and the appraisal committee had a single external expert (was this enough?) and patient advocate (NICE, 2002). The published guidance advocates the use of 5-fluorouracil and folinic acid (5FU/FA) initially, reserving single-agent irinotecan for when this treatment fails. Oxaliplatin was recommended for use in the very few cases where inoperable liver metastases can potentially be made operable with chemotherapy. The use of raltitrexed was not recommended at all.

We are therefore left with the unpalatable situation of following guidance that limits us to using a 40-year-old drug as first-line treatment in the majority of patients with advanced CRC. This guidance is at odds with the Oncologic Drugs Advisory Committee of the US Food and Drug Administration (FDA), which approved the first-line use of combined irinotecan and 5FU/FA (Ir.5FU/FA) in March 2000. It is also at odds with much of Europe, where either oxaliplatin or irinotecan are regularly used in combination with 5FU/FA in this group of patients. In addition, during the period of consultation prior to the final Guidance, many purchasers withheld funding for these drugs and therefore patients in NHS Trusts were potentially denied a clinically effective treatment for approximately 18 months.

The evidence for the use of Ir.5FU/FA in the first-line treatment of patients with advanced CRC is very compelling. Two large randomised trials have been published (Douillard *et al*, 2000; Saltz *et al*, 2000) that show a significant survival benefit when patients receive an Ir.5FU/FA combination compared to 5FU/FA alone. More than 1000 patients were entered into these studies and, amongst patients randomised to receive ‘standard’ 5FU/FA, there was significant ‘cross-over’ to irinotecan-based therapy upon disease progression (31% and 56% in the two studies, respectively). None-the-less there was still a 3-month survival benefit in the group of patients that initially received Ir.5FU/FA. This, along with historical 5FU data, suggests that the survival benefit would probably be even greater than 3 months if ‘cross-over’ were not allowed. It also suggests that giving irinotecan ‘up-front’ is advantageous, given that patients in this arm fared better even though a considerable number of patients in the control arms also crossed-over to receive irinotecan after failing 5FU/FA. NICE also evaluated other studies that had been published as abstracts including the FOLFOX/FOLFIRI data (Tournigand *et al*, 2001). This study randomised patients to receive either oxaliplatin and 5FU/FA (Ox.5FU/FA) or Ir.5FU/FA initially, crossing over to receive the other arm when the disease progressed. The response rate to both arms was approximately 57% and the median survival exceeds that seen in any previous advanced CRC studies (approx. 21 months in both treatment arms – data presented later at the American Society of Clinical Oncology (ASCO), San Francisco, 2001). Publication of this important paper is expected imminently. To summarise, the evidence for using irinotecan ‘up-front’ in patients with advanced CRC is very strong and importantly, all the available data points in the same direction: increased survival. We feel therefore, that it is perverse that NICE have come to this conclusion given the weight of the data supporting the use of irinotecan in this situation.

Combinations of Irinotecan with 5FU/FA are well tolerated and a patient's quality of life (QoL) is no worse than with 5FU/FA (de Gramont regimen) alone (Douillard *et al*, 2000). However, single agent irinotecan, as recommended by NICE, is given at approximately twice the dose and the prevalence of alopecia, and grade III/IV nausea (15% *vs* 2.1%), diarrhoea (22% *vs* 13.1%) and vomiting (14% *vs* 2.8%) is increased compared to the 2-weekly irinotecan/de Gramont schedule commonly used in the UK (Cunningham *et al*, 1998; Douillard *et al*, 2000).

If Irinotecan and 5FU/FA were given ‘up-front’ to patients with advanced CRC as recommended by the FDA, they would receive a single period of ‘active treatment’ until disease progression. Upon disease progression, they may then have the opportunity to enter appropriate clinical trials or receive best supportive care (BSC). If we follow the NICE guidance, patients would receive 5FU/FA until disease progression followed by single agent irinotecan if they are still fit enough. This would lead to two periods of ‘active’ treatment no doubt amounting to 6 to 12 months of chemotherapy, when patients would receive more intensive follow-up than if they received BSC. This is expensive in terms of staff time and more importantly patient time as well as expensive in monetary terms. More out-patient visits will be required, more demand on pharmacy services will be generated, out-patient congestion will be increased for longer periods, more CT scans, ultra-sounds and chest X-rays will be required and there will be an increased burden on the hospital pathology departments. In this issue, Cunningham *et al* support this view and state that their data for cost effectiveness and clinical evidence ‘strongly support the use of irinotecan and 5FU/FA in the setting of first line therapy of metastatic CRC’ (Cunningham *et al*, 2002). They have shown a cost-effectiveness ratio per life year gained (LYG) of » 14 794, which compared favourably to a range surmised from a recent Department of Health review. The cost-effectiveness of different 5FU regimens is also discussed in this issue (see Hale *et al*, 2002).

The oxaliplatin studies (de Gramont *et al*, 2000; Giacchetti *et al*, 2000) also suffered from the crossover effect from the control arms to the oxaliplatin-based therapy on disease progression. In particular, the randomised study by de Gramont *et al* (2000) showed a much higher than expected median overall survival in the control 5FU/FA arm because of the crossover of these patients to the oxaliplatin-containing arm or because they also received irinotecan at some point. If crossover was not allowed, a significant survival benefit may well have been achieved. What is now becoming evident is that the use of all three drugs (Ir, Ox, 5FU) in different combinations and sequencing leads to improved patient survival (Tournigand *et al*, 2001). If we follow the NICE guidelines, then the survival of patients with advanced CRC in England and Wales will no-doubt be reduced compared to the rest of Europe and USA, since our freedom to prescribe these newer agents will be restricted.

Very few patients have inoperable liver metastases at presentation, that can be down-staged enough with chemotherapy to make them operable. Therefore the recommendation by NICE that Ox.5FU/FA combination chemotherapy should be used in this scenario will only benefit a very small number of patients. The guidance is only based on retrospective data from two studies (Adam *et al*, 2001; Bismuth *et al*, 1996; Giacchetti *et al*, 2000). This recommendation is therefore surprising given that they were unable to recommend first-line Ir.5FU/FA combinations with evidence based on two large prospective RCT's. NICE have also failed to adequately define the type of patients for which this treatment would be appropriate. The characteristics of such patients will need to be clearly defined at a multidisciplinary level, particularly in conjunction with hepatobiliary surgeons. None-the-less this unexpected recommendation is welcomed, and will allow us to use oxaliplatin in this setting.

The use of raltitrexed in patients with advanced CRC was also not recommended by NICE. We feel that this decision is again wrong given that patients with significant heart disease will be denied an effective treatment. 5-Fluorouracil should be used with caution or should even be contraindicated in patients with troublesome arrhythmias and ischaemic heart disease. At present the only effective alternative is raltitrexed and we feel that this drug should be made available and used with caution in this particular situation. NICE may maintain that they only produce ‘guidance’, however, their documents are often taken literally by funding bodies and so money for raltitrexed, for example, is unlikely to be made available despite clinical need.

We therefore need to ask, ‘Why hasn't NICE followed North America and Europe's lead and recommended more widespread use of these drugs in England and Wales?’ We believe that cost was the main issue. They have essentially restricted the use of irinotecan by accepting that only 75% of patients (possibly an overestimate) will still be fit enough to receive this drug after first-line 5FU/FA (NICE, 2002). Therefore, 25% of patients who were initially deemed fit enough to receive a first-line combination of Ir.5FU/FA will in fact be denied this therapy because their disease has progressed and fitness has deteriorated. Rationing patient's treatment in this manner is not the way to save money. Additionally, the use of two active periods of treatment as outlined above is likely to prove this strategy a ‘false economy’.

One of the government aims is to remove ‘post-code prescribing’. NICE have gone some way to achieving this by allowing us to prescribe irinotecan for patients who fail first-line 5FU/FA. This is certainly a welcome improvement for hospitals not previously able to prescribe this drug at all. However, NICE would have done even better if they had allowed us more freedom to prescribe these newer agents appropriately, in the same manner as in North America and much of Europe. We would then get closer to achieving the aims of the National Cancer Plan. For now, NHS patients in England and Wales will have to accept inferior treatment and a reduced life expectancy.

A House of Commons Science and Technology Committee Special Report on cancer research was published on the 20th of March 2002. The report claims that there is insufficient funding from the Department of Health for cancer research. This may not surprise many of us since it has long been felt that we rely too much on the various charities and the pharmaceutical industry. To continue with internationally accepted research, we need to have a control arm that is acceptable to the rest of the world. If North America are using irinotecan regularly and Europe are using either this or oxaliplatin, do we continue to simply use 5FU/FA? Research using just a 5FU/FA-control arm will no longer be considered relevant by others outside the UK. Gaining funding will no doubt be harder, considering that these bodies, quite rightly, want to fund research that is up-to-date and internationally accepted.

We have yet to mention the patient's perspective. Given that informed consent and patient choice are becoming increasingly paramount, it would be interesting to hear their view if all of the facts were presented to them. Would they prefer the NICE strategy or the treatment approved by the FDA? – One can only speculate, but we think the answer would be obvious.

NICE is due to review this guidance in 2005 when data from the MRC CRO8 (FOCUS) study becomes available. Given that the results from this study are unlikely to dramatically alter the balance of information presently available, this seems tardy. By that time, the use of these newer agents in North America and Europe will have become established and we will trail even further behind in the treatment of advanced CRC. Results will also become available from a number of adjuvant colon cancer studies that have recently closed (X-ACT, MOSAIC, PETACC-3). If the outcomes are favourable, then it is possible that these trials may prove that these newer agents are beneficial in this situation, even though we are unable to prescribe them for patients with advanced CRC.

The 5-year survival figures for the UK are already extremely poor compared to Europe and North America (41, 47 and 63% respectively) (National Statistics (2000); Office for National Statistics, 2000). In a typically political statement, one of the aims of the National Cancer Plan is to ‘never fall behind again’. However, in the case of CRC, it is perhaps difficult to fall behind when we haven't even reached the baseline from which to fall behind from!! Hopefully some of the money announced in the recent budget can be used to provide modern, effective, chemotherapy for patients with this awful disease. Then perhaps we can at last compete with the rest of the world.
